# Fire and Herbivory as Architects of Mediterranean Biodiversity

**DOI:** 10.1002/ece3.72534

**Published:** 2025-11-20

**Authors:** Marion Lestienne, Pauline Saurat, Christel Vidaller, Gwendal Mouden, Andy Hennebelle, Lisa Bajolle, Bérangère Leys

**Affiliations:** ^1^ IMBE Aix Marseille Université, Avignon Université, CNRS, IRD Aix‐en‐Provence France; ^2^ AMAP University of Montpellier, CIRAD, CNRS, INRAE, IRD Montpellier France

**Keywords:** coprophilous spores, disturbance ecology, fire regime, Holocene, Mediterranean ecosystems, palaeoecology, pastoralism, vegetation dynamics

## Abstract

Reconstructing the long‐term interactions between fire and herbivory is essential to understand how Mediterranean vegetation has historically responded to disturbance regimes which is a critical step for informing current biodiversity and fire management strategies. We reconstructed 8000 years of vegetation composition, habitat combustibility, and herbivore density in southern France using pollen data and coprophilous fungal spores. We show that periods of high‐herbivore density systematically co‐occur with open, highly flammable habitats, and significantly correlate with elevated palynological richness. Conversely, cooler and wetter climatic phases promoted the development of closed‐canopy, low‐combustibility forests, which consistently exhibited lower biodiversity levels. In recent centuries, a documented decline in mammal herbivory pressure has coincided with the expansion of fire‐prone vegetation types such as garrigue and green oak coppice, exacerbating landscape combustibility under climate change. Our 8000‐years reconstruction highlights herbivory as a persistent and quantifiable driver of habitat openness, heterogeneity, and fire regime modulation. *Synthesis*: Our study demonstrates that herbivores and fire have jointly shaped Mediterranean biodiversity over millennia. These findings highlight the need to reintroduce or maintain herbivory as a management tool in fire‐prone Mediterranean ecosystems.

## Graphical abstract

Our 8000‐year palaeoecological reconstruction from southern France reveals that herbivores and fire have jointly maintained open, heterogeneous, and biodiverse Mediterranean landscapes. Periods of high‐herbivore density consistently coincided with flammable, species‐rich habitats, while declines in grazing promoted closed forests with lower diversity. These results highlight herbivory as a key ecological driver and a potential management tool for sustaining biodiversity and reducing fire risk in Mediterranean ecosystems.

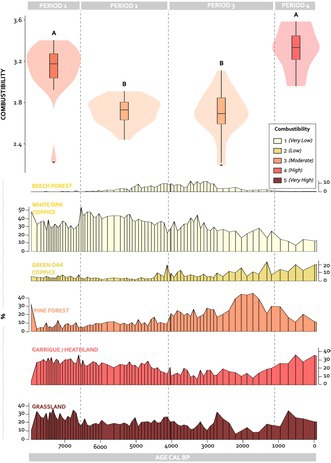

## 1 Introduction

The Mediterranean region is among the most species‐rich biomes on Earth (Myers et al. 2000). Its remarkable biodiversity reflects a long history of interactions between disturbances (i.e., fires and herbivores), climate variability, anthropogenic activities, and land uses such as sylvo‐pastoralism (Colombaroli et al. 2007; Lestienne, Jouffroy‐Bapicot, et al. 2020). However, Mediterranean ecosystems are increasingly under pressure from climatic shifts and human impacts, making it essential to understand their resilience and dynamics in order to identify thresholds of ecological change and inform strategies for sustaining biodiversity, ecosystem functions, and traditional land‐use practices (Colombaroli and Tinner 2013; Giannakopoulos et al. 2005).

Disturbances such as fire and herbivory have long acted as major organizing forces in Mediterranean ecosystems. In particular, fire (sustained by warm, dry summers and dense, flammable vegetation) has shaped plant communities over millennia, favoring fire‐adapted species and maintaining open habitats (He et al. 2019; Keeley 2009). Fire‐adapted species and open habitats owe their persistence to the fire regime, which has helped maintain the mosaic of landscapes characteristic of the region (Leys et al. 2018; Mouillot and Field 2005). Although fire is a well‐established disturbance acting on Mediterranean vegetation dynamics and landscape structure, herbivores have been demonstrated to play a key role in Mediterranean landscape dynamics (Berger et al. 2019; Lee et al. 2022). Both wild herbivores and domesticated livestock have shaped vegetation patterns over millennia. Rather than simply limiting forest development, they could promote plant diversity by reducing woody species dominance and maintaining open, heterogeneous habitats that support a wide range of taxa (Lestienne, Jouffroy‐Bapicot, et al. 2020; Sandom et al. 2014; Zeder 2008). While the role of mammal herbivory by domestic livestock in pastoral systems is well documented (Jouffroy‐Bapicot et al. 2016; Lestienne, Jouffroy‐Bapicot, et al. 2020), the ecological impact of wild herbivores remains poorly understood. Yet, distinguishing between these two sources of herbivory is essential to assess how vegetation dynamics responded to herbivore pressure before the onset of agriculture and in contexts where pastoralism was absent or limited (Behrensmeyer 2015). In addition, the combined impacts of both herbivores and fire are poorly reported due to the lack of records for both disturbances in this area (Lestienne, Jouffroy‐Bapicot, et al. 2020; Smit and Coetsee 2019). This lack of integrated knowledge limits our understanding of how fire and herbivory interact to shape vegetation structure and biodiversity. Such understanding is crucial for anticipating ecosystem responses to future land‐use changes and climate‐driven shifts in fire regimes and herbivore populations, especially in Mediterranean landscapes where both pressures are intensifying. To disentangle the long‐term ecological roles of fire and herbivory, we tested the hypothesis that herbivore density has historically covaried with vegetation openness, habitat combustibility, and biodiversity. Specifically, we expected that periods of high‐herbivore presence would correspond to open habitats (defined here as landscapes with reduced tree and shrub cover and a predominance of herbaceous vegetation; e.g., Morales‐Molino et al. 2021; Tinner et al. 2005), which, despite a local reduction of biomass due to mammal herbivory, tend to maintain fine and continuous fuels that dry rapidly and thus remain prone to fire under warm and dry Mediterranean conditions. Conversely, low herbivory would align with more closed, low‐diversity forest stages.

We evaluated this using an 8000‐year palaeoecological record from the Crau Plain in southern France. This region, combining Mediterranean steppe and oak woodlands, offers a rare opportunity to investigate disturbance–diversity relationships in a system where open habitats have persisted over millennia. The Crau thus serves as a representative case to infer how herbivory and fire have jointly shaped biodiversity patterns in Mediterranean landscapes. By combining multi‐millennial records of vegetation structure and herbivore activity, we test how fire and herbivory have jointly shaped biodiversity and vegetation composition in Mediterranean ecosystems. This approach allows us to explore whether these two disturbances acted in synergy or whether their effects were independent or antagonistic. Understanding these long‐term interactions is essential not only for Mediterranean ecology but also for broader questions in disturbance ecology, biodiversity maintenance, and nature‐based fire management strategies. To assess how vegetation structure influenced fire behavior over time, we grouped plant taxa into habitat types based on their combustibility (Trabaud 1971). This functional approach allows us to evaluate how shifts in vegetation structure, such as the dominance of open versus closed habitats, influenced habitat combustibility and how these changes interacted with herbivore pressure to shape biodiversity levels and vegetation turnover through time. This approach will enhance our understanding of the processes underpinning Mediterranean biodiversity and inform strategies for sustainable ecosystem management under future environmental pressures.

## 2 Materials and Methods

### 2.1 Study Area

**FIGURE 1:**
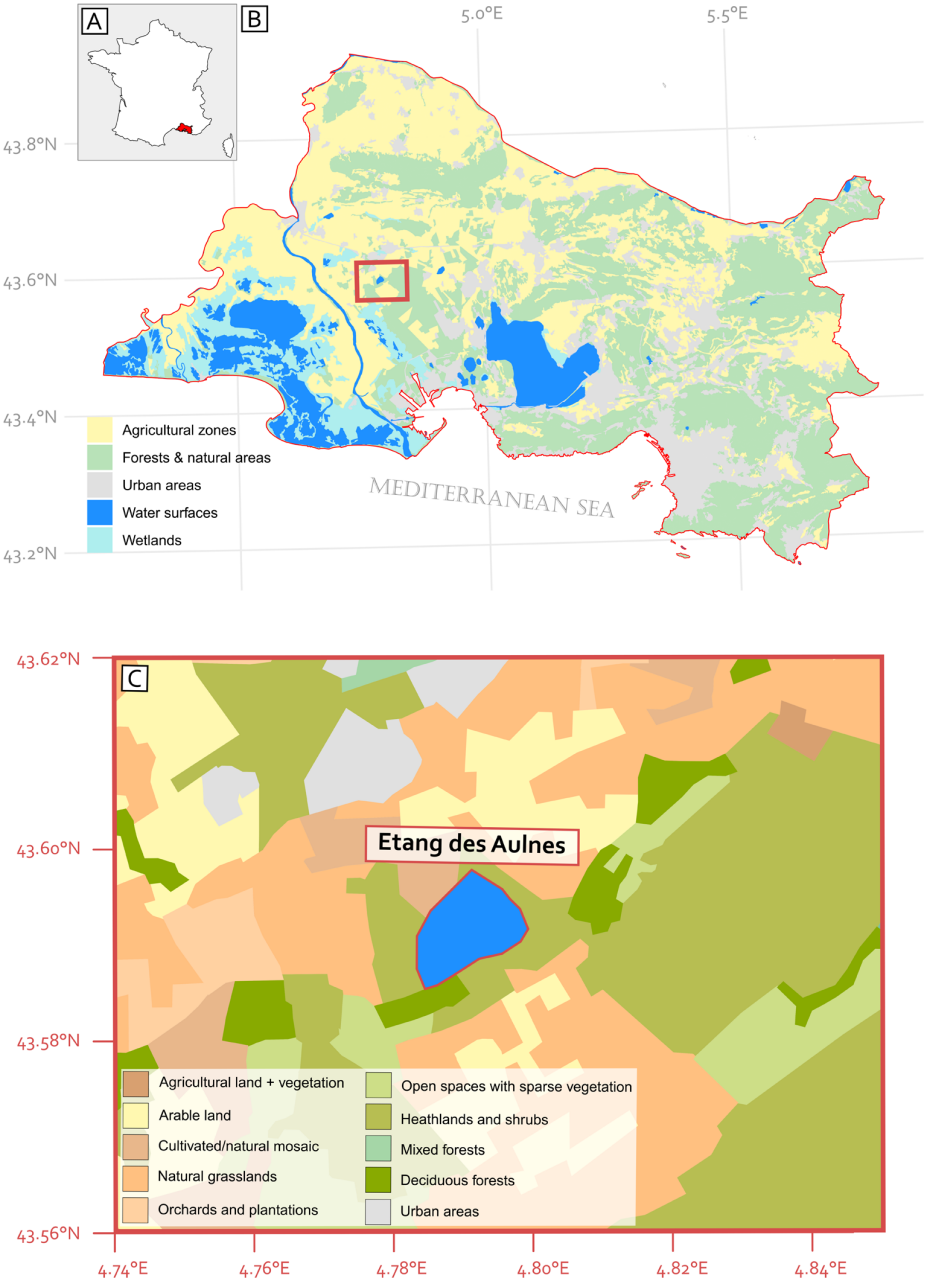
Study area and land cover classification. (A) Map showing the study region in France, with the studied area highlighted in red. (B) Land cover classification of the department based on the CORINE Land Cover 2018 dataset, showing the main land cover groups: artificial surfaces (e.g., urban fabric, industrial and commercial areas, road networks), agricultural areas (e.g., arable land, permanent crops, pastures, heterogeneous agricultural landscapes), forest and seminatural areas (e.g., broad‐leaved and coniferous forests, natural grasslands, shrublands), wetlands (e.g., inland marshes, peat bogs), and water bodies (e.g., rivers, lakes, reservoirs). The study site is indicated by a red rectangle. (C) Detailed land cover map of the study area, providing a finer‐scale classification of habitat types within the site and their association with the broader CORINE Land Cover categories, allowing for a more precise understanding of local landscape heterogeneity.

This study examines a pond located in the Crau plain, the Étang des Aulnes (43°35′33″ N, 4°47′23″ E), situated in the Saint‐Martin‐de‐Crau area in southern France (Figure 1). The Crau plain is characterized by a topography of plateaus and hills, with a maximum altitude of 142 m. This plain is a fossilized riverbed, with 50% of its surface covered by siliceous stones. A limestone layer overlays the soil, which has a thickness of approximately 40 cm and lies above an impermeable conglomerate layer that ranges from 1 to 5 m deep. This conglomerate prevents plant roots from accessing the alluvial water table (Colomb and Roux 1978). Currently the Crau plain experiences a Mediterranean climate, with hot, dry summers (June–September) and mild, wet winters (November–February) (Magny et al. 2007). The Crau plain hosts diverse ecosystems, including an alluvial forest and a steppe composed of Poaceae species such as *Brachypodium retusum* and *Thymus vulgaris* (Triat‐Laval 1979).

### 2.2 Chronology

A core of 4 m of sediments was extracted from the Aulnes pond in 2023 and 9 ^14^C dates from macroplant remains and bulk sediments helped establish the depth–age relationships, modeled with a Bayesian model using the “rbacon” package (Blaauw and Christen 2011) (for details, see Table S1). For the entire sequence, the reconstruction spans ca. 7960 years (min: 7600; max: 8540 years cal. BP), given a median resolution of 103 years per sample.

### 2.3 Pollen and Coprophilous Fungal Spore's Analysis

Pollen analysis has been used to reconstruct past vegetation dynamics (Birks and Tinner 2016), while coprophilous fungal spores serve as proxies for herbivore presence and abundance (van Geel et al. 1989; Table S2). Subsamples (2 cm^3^) were collected at intervals of 4 cm, providing a temporal resolution between the sampling year and ca 7960 years cal. BP (min: 7600; max: 8540 years cal. BP). Lycopodium marker tablets (Stockmarr 1971) were added to each sample for estimation of the pollen concentration (grains cm^−3^) and influx (grains cm^−2^ year^−1^). Pollen grains and spores were extracted following standard techniques (Faegri and Iversen 1989), in which successive suspensions in 40% hydrofluoric acid (HF), 10% hydrochloric acid (HCl), and acetolysis were used to remove silicates, carbonates and cellulose respectively. The pollen grains and spores were counted under a light microscope at ×400 magnification. Pollen identification followed using pollen keys and photo collections (Punt 1984; Reille 1992; Beug 2004). Coprophilous fungal spores were identified from the Non‐Pollen Palynomorph Image Database (Shumilovskikh et al. 2022; Table S3).

An average of 450 pollen grains of terrestrial species was counted for each sample, with a maximum of 788 grains and a minimum of 92 grains for the deepest sample. Pollen percentage was calculated based on the pollen sum excluding (semi)aquatic pollen and coprophilous fungal spores. Pollen diagram was constructed using the R package “rioja” (Juggins 2024). A constrained sum‐of‐squares cluster analysis (CONISS) (R Core Team 2019; Simpson 2007) was used to identify pollen assemblage zones, and the significance of each zone was tested with the broken stick model (Bennett 1996) using the “rioja” R package (Juggins 2024).

Each pollen taxon was associated with a specific species linked to a habitat type based on Trabaud's classification (Trabaud 1971), vegetation surveys (personal communication from C. Vidaller, unpublished data) and Mediterranean plant lists established for the area with Word Flora online (Elliott et al. 2023). This approach allowed the grouping of pollen taxa into ecological categories reflecting the combustibility and vegetation dynamics of the identified habitats (see Table S2 for details). When a species could not be directly assigned to a habitat in Trabaud's classification, it was grouped into a separate “other” category (28.52% of the total pollen influx). The majority of the taxa in this category represent species associated with riparian or floodplain vegetation (e.g., *Alnus* sp., *Abies* sp., *Ulmus* sp.), which do not align directly with the habitats defined by Trabaud.

To quantify the combustibility of each habitat, we followed Trabaud's ecological framework (Trabaud 1971, 1974), which links plant community structure to potential fire behavior. In his classification, combustibility reflects the capacity of a vegetation type to sustain and propagate fire once ignition occurs, integrating parameters such as biomass quantity and continuity, stratification (vertical fuel arrangement), dominant species composition, and volatile compound content. Each habitat type was thus assigned a relative combustibility score ranging from 1 (very low) to 5 (very high), corresponding respectively to closed, humid, and low‐combustibility forests (e.g., beech stands) and open, xeric, highly flammable formations (e.g., garrigue). The scoring is based on Trabaud's field‐derived hierarchy of Mediterranean vegetation combustibility, refined by subsequent experimental and cartographic studies (Trabaud 1971, 1974).

For each sample, the mean habitat combustibility was calculated as a pollen‐influx‐weighted average of the discrete combustibility classes (1–5) assigned to each habitat type, following Trabaud's classification using the formula:

$$\text{Mean combustibility}=\frac{\sum \left(\text{influx}\;\left(i\right)\times \text{combustibility}\;\left(i\right)\right)}{\text{total influx}}$$

where *i* corresponds to the habitat type. This weighting approach yields a continuous index reflecting the overall combustibility of the surrounding vegetation.

This method allows us to move beyond a purely taxonomic view of vegetation and to interpret palynological assemblages in terms of fire ecology and landscape combustibility, providing a functional perspective on vegetation–fire–herbivory interactions through time.

### 2.4 Diversity Analysis

To assess the biodiversity changes in the surroundings of the sample site, pollen diversity was measured using richness (Birks and Line 1992) and evenness (Hurlbert 1971) to encompass all diversity dimensions. Palynological richness (PRI) represents the α‐diversity corresponding to the expected number of taxa found in samples of equal size as estimated by rarefaction analysis. It is a robust method used in many palaeoecological studies in Europe (Colombaroli and Tinner 2013; Lestienne, Jouffroy‐Bapicot, et al. 2020; Lestienne et al. 2023). PRI was calculated by using the “Vegan” package on all pollen taxa (Dixon 2003) of the statistical software R (R Core Team 2019) and a constant pollen sum, which was standardized on the minimum pollen sum (*n* = 92). Although the representativeness of pollen to portray the plant richness has been questioned over decades overall pollen richness is considered a good indicator of vegetation richness (Birks et al. 2016). While it is a valuable measure of long‐term biodiversity changes (Colombaroli and Tinner 2013; Lestienne, Jouffroy‐Bapicot, et al. 2020), these results must be interpreted carefully (Weng et al. 2006).

The probability of interspecific encounters (PIE) was used as an index of evenness (Hurlbert 1971). This index gives the probability of two randomly sampled pollen grains from a given habitat type representing two different sets of species. To assess possible alteration of PRI due to evenness influence, we calculated evenness‐detrended palynological richness (DPRI) (Colombaroli and Tinner 2013). PRI is regarded as being unaffected by palynological evenness if both PRI and DPRI show similar trends (Colombaroli and Tinner 2013).

The palynological turnover has been computed based on an improved algorithm (R package “R‐Ratepol”) for estimating rates of change (RoCs) for palaeoecological time series (Mottl et al. 2021). RoCs were calculated as chi‐squared dissimilarities (dissimilarity_coefficient = “chisq”) between temporally adjacent working units. These working units were defined using a moving window approach (working_units = “MW”) applied to 200‐year time bins, shifted five times to capture local variability (bin_size = 200, number_of_shifts = 5). Prior to dissimilarity calculation, assemblage data were smoothed using age‐weighted averaging (smooth_method = “age.w”) to account for unequal sampling intervals while retaining ecological signals. Compositional matrices were standardized to the lowest total count across all samples (standardize = TRUE, n_individuals = min_count) to avoid bias due to differences in sampling intensity. A total of 100 randomizations (rand = 100) were performed to account for stochasticity introduced by resampling. Rates of change were finally rescaled to represent dissimilarity per 200 years (time_standardisation = 200). Significant RoC peaks were detected using a nonlinear trend detection method (sel_method = “trend_non_linear”), allowing identification of abrupt transitions in community structure over time (terms under brackets correspond to parameters used to run the function).

Linear models were applied to assess relationships between biodiversity metrics and herbivory indicators only when the residuals conformed to a normal distribution, ensuring that the assumptions of linear regression were met. However, since the original variables often deviated from normality, nonparametric Spearman's rank correlation tests were additionally performed to evaluate monotonic associations without relying on distributional assumptions. This dual approach allowed us to capture both linear trends and robust rank‐based correlations.

## 3 Results

### 3.1 Chronology and Age–Depth Model

**FIGURE 2:**
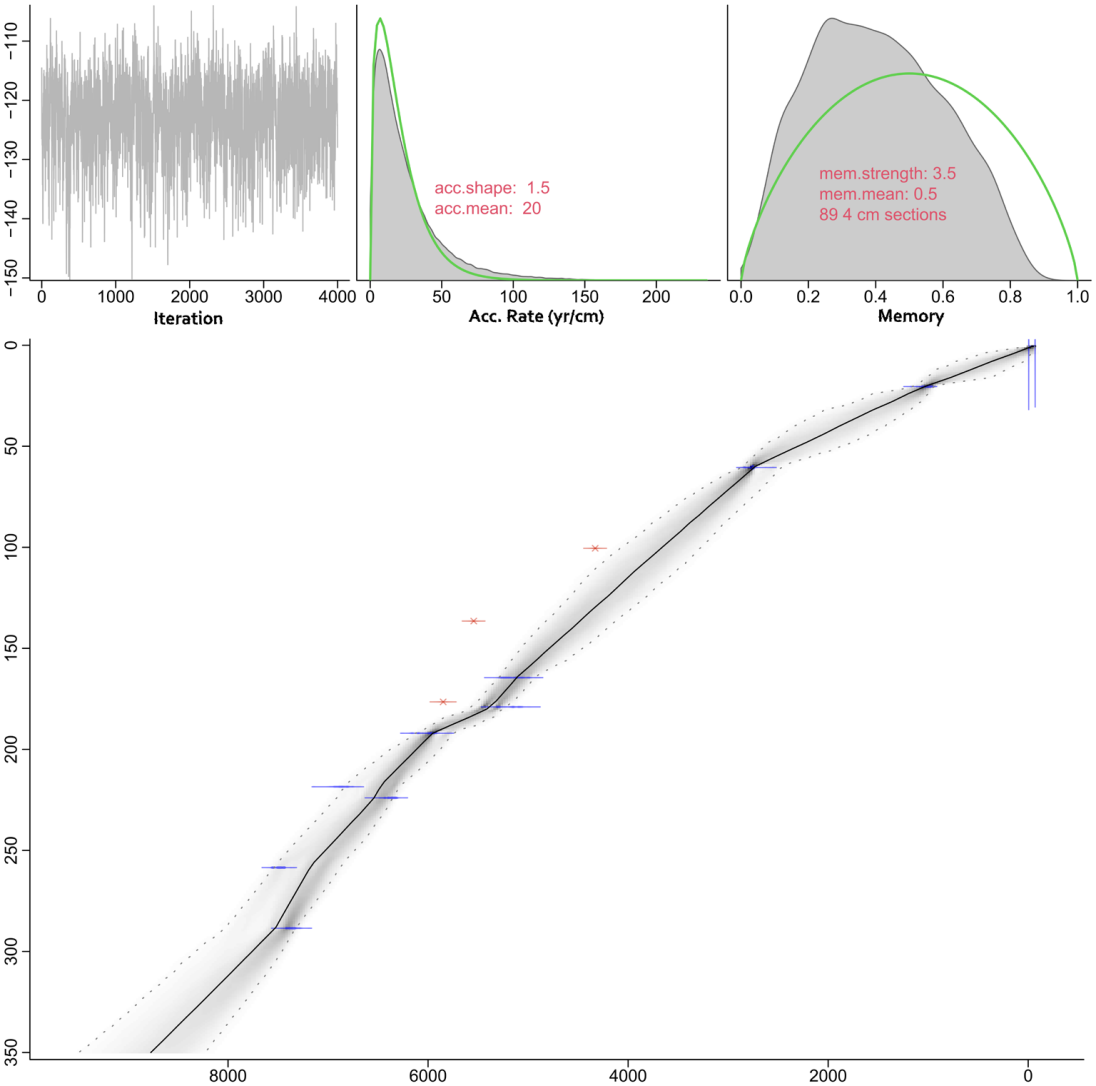
Age–depth model of etang des Aulnes computed with “rbacon” and based on nine AMS radiocarbon dates; red dots correspond to ^14^C dates not taken into account in the age–depth model. The shaded area corresponds to the 95% confidence interval, and the dotted red curve represents the mean model.

The model spans the last ca. 7960 years, with calibrated ages ranging from 7605 to 8538 years cal. BP (Figure 2). Three of the dates were excluded from the model as they contained not enough material to provide robust dating measurements and are marked in red. The mean accumulation rate is estimated at 20 years per centimeter, with an associated shape parameter of 1.5. The memory strength of the model is 3.5, with a mean memory of 0.5, indicating moderate autocorrelation between adjacent sections. The lower panel shows the age–depth relationship, with the black curve representing the mean model and the gray envelope indicating the 95% confidence interval. Blue bars represent the calibrated 2*σ* ranges for included dates. Depth intervals between dated levels show variable accumulation rates, with tighter confidence intervals in well‐constrained sections and broader uncertainties in undated intervals.

### 3.2 Habitat Composition and Combustibility

**FIGURE 3:**
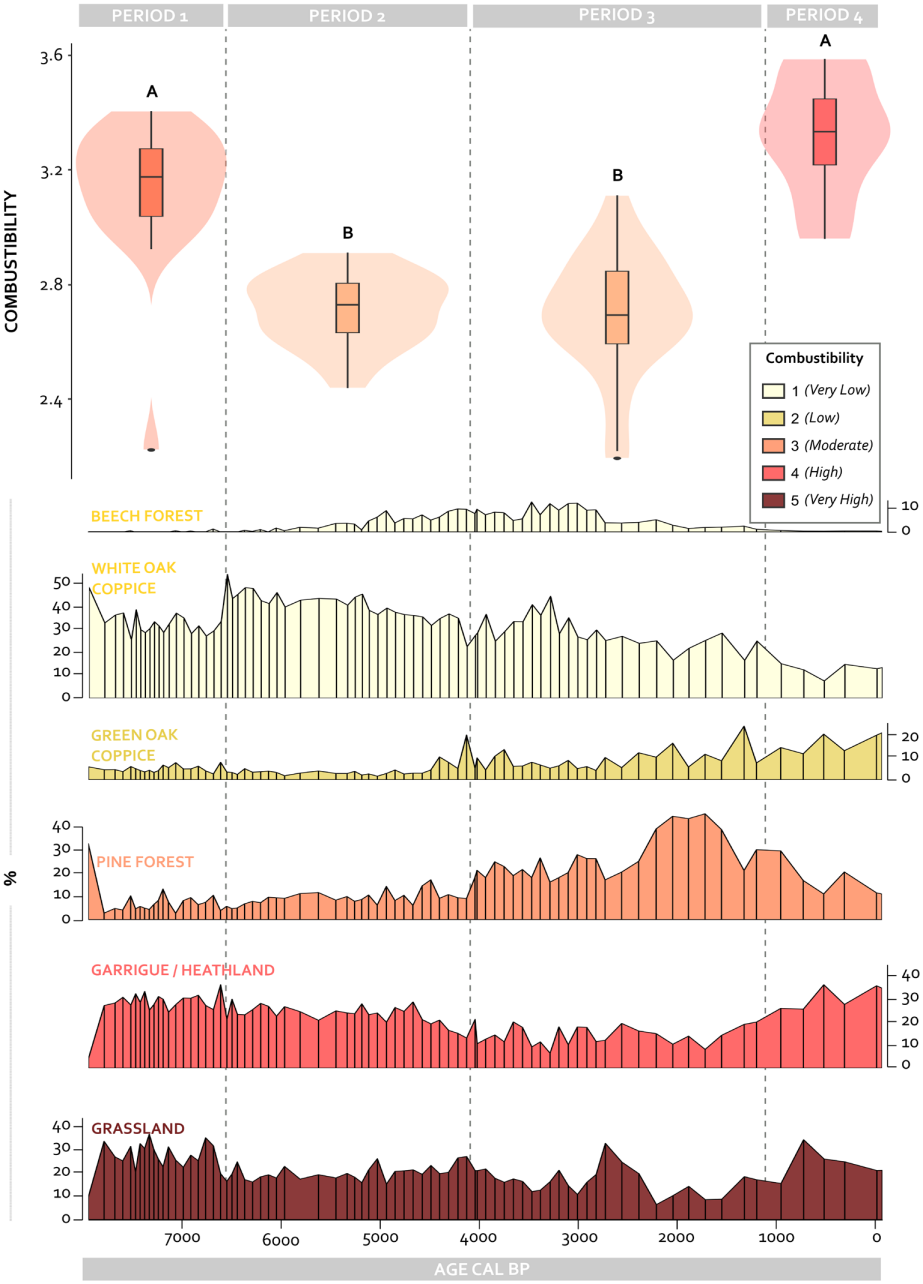
Temporal evolution of habitats in the Crau plain, grouped according to Trabaud's (1971) habitat classification. Boxplots and violins represent combustibility across four periods, as inferred from the pollen data. Below the boxplots, the influx of pollen taxa is shown for each habitat type: Beech Forest, White Oak Coppice, Green Oak Coppice, Pine Forest, Garrigue/Heathland, and Grassland. Pollen percentages values illustrate the relative abundance of taxa for each habitat. Habitat groupings were established based on their ecological and combustibility characteristics as defined by Trabaud's classification (see Table S2 for details). Combustibility is classified into five levels based on vegetation characteristics and fire behavior. Class 1 (Very Low) represents habitats with low combustibility and limited fire propagation, typically found in humid or temperate environments like beech forests or white oak coppices. Class 2 (Low) includes vegetation that burns under specific conditions, such as prolonged drought, with moderate fire spread, as seen in green oak coppices. Class 3 (Moderate) corresponds to habitats with higher combustibility and faster fire spread, such as pine forests, where resinous materials enhance combustion. Class 4 (High) is characterized by highly flammable vegetation, rich in volatile compounds like essential oils or resins, leading to intense fires, typical of garrigue and heathlands. Class 5 (Very High) includes vegetation that is extremely flammable and burns explosively, with rapid fire propagation, as seen in dry grasslands dominated by fine, dry biomass. A nonparametric Mann–Whitney *U* test was conducted to compare mean combustibility between two periods. Results are displayed within the figure as pairwise comparisons using letters to indicate statistically significant differences. Groups sharing the same letter are not significantly different. The mean combustibility for periods 1–4 is 3.12, 2.71, 2.69, and 3.30, respectively. Data do not follow a normal distribution. Significant differences were determined using a nonparametric Mann–Whitney *U* test, with the following *p* values: periods 1 versus 2 (*p* value = 1.754e‐08), periods 1 versus 3 (*p* value = 1.004e‐08), periods 2 versus 4 (*p* value = 1.281e‐06), and periods 3 versus 4 (*p* value = 1.677e‐06). Periods 1 versus 4 (*p* value = 0.1036) and periods 2 versus 3 (*p* value = 0.9324) show no significant differences.

Pollen data reveal shifts in habitat composition over the last ~8000 years, with taxa grouped into six habitat types: beech forest (e.g., *Fagus sylvatica* ), white oak coppice (e.g., *Quercus pubescens*), green oak coppice (e.g., *Quercus ilex* ), pine forest (e.g., *Pinus halepensis* ), garrigue/heathland (e.g., *Plantago lanceolata* ), and grassland (e.g., *Brachypodium* sp.). Each habitat was assigned a combustibility score from 1 (very low) to 5 (very high) based on empirical observations of Mediterranean vegetation combustibility, including parameters such as biomass structure, volatile compound content, moisture retention, fire propagation speed, and post‐fire regeneration strategies (Trabaud 1971, 1974). Combustibility values differ significantly between time periods. Period 1 shows a mean combustibility of 3.12, followed by 2.71 in Period 2, 2.69 in Period 3, and 3.30 in Period 4. Pairwise comparisons indicate significant differences between Periods 1–2, 1–3, 2–4, and 3–4 (*p* < 0.0001), with no significant difference between Periods 1–4 (*p* = 0.1) or 2–3 (*p* = 0.9) (Figure 3; Figures S1 and S2 for more details).

Combustibility fluctuates markedly throughout the Holocene, reflecting shifts in vegetation composition and structure. During the early Middle Holocene (approximately 8000–6500 years cal. BP), combustibility is high (mean = 3.12, SD = 0.25, SE = 0.06), which corresponds to Class 3–4 vegetation in Trabaud's combustibility scale (moderately to highly flammable) (Trabaud 1971, 1974). This is the highest average combustibility value observed across the sequence (see Figure 3). It is associated with a wide distribution of combustibility scores, reflecting the coexistence of both moderate and highly flammable habitats. This pattern is driven by the white oak coppice and a substantial increase in grassland cover, which significantly contributes to the overall combustibility. Pine forest is present but less abundant, while beech forest and green oak coppice were nearly absent. Between 6500 and 4000 years cal. BP (mid to late Middle Holocene), combustibility decreases (mean = 2.71, SD = 0.13, SE = 0.03) as closed‐canopy, low‐combustibility habitats become more prevalent. White oak coppice remains dominant, but beech forest increases significantly, reaching its peak during this interval, whereas green oak coppice and pine forest remain relatively limited. This trend continues during the Late Holocene (approximately 4000–1000 years cal. BP), with mean estimated combustibility remaining low (mean = 2.69, SD = 0.21, SE = 0.04), supported by high proportions of beech forest and the continued decline of white oak coppice. At the same time, pine forest becomes more abundant, and green oak coppice gradually expands. A marked shift occurs in the most recent period (approximately 1000 years cal. BP to the present), when combustibility rises sharply (mean = 3.30, SD = 0.22, SE = 0.08), returning to levels comparable to those of the early Middle Holocene. This increase coincides in time with the dominance of open, highly combustible habitats such as grassland and garrigue or heathland, followed by green oak coppice. Pine forest persists in moderate amounts, while low‐combustibility forests, particularly white oak and beech, experience a sharp decline or near disappearance.

### 3.3 Diversity Patterns and Herbivore Indicators

**FIGURE 4:**
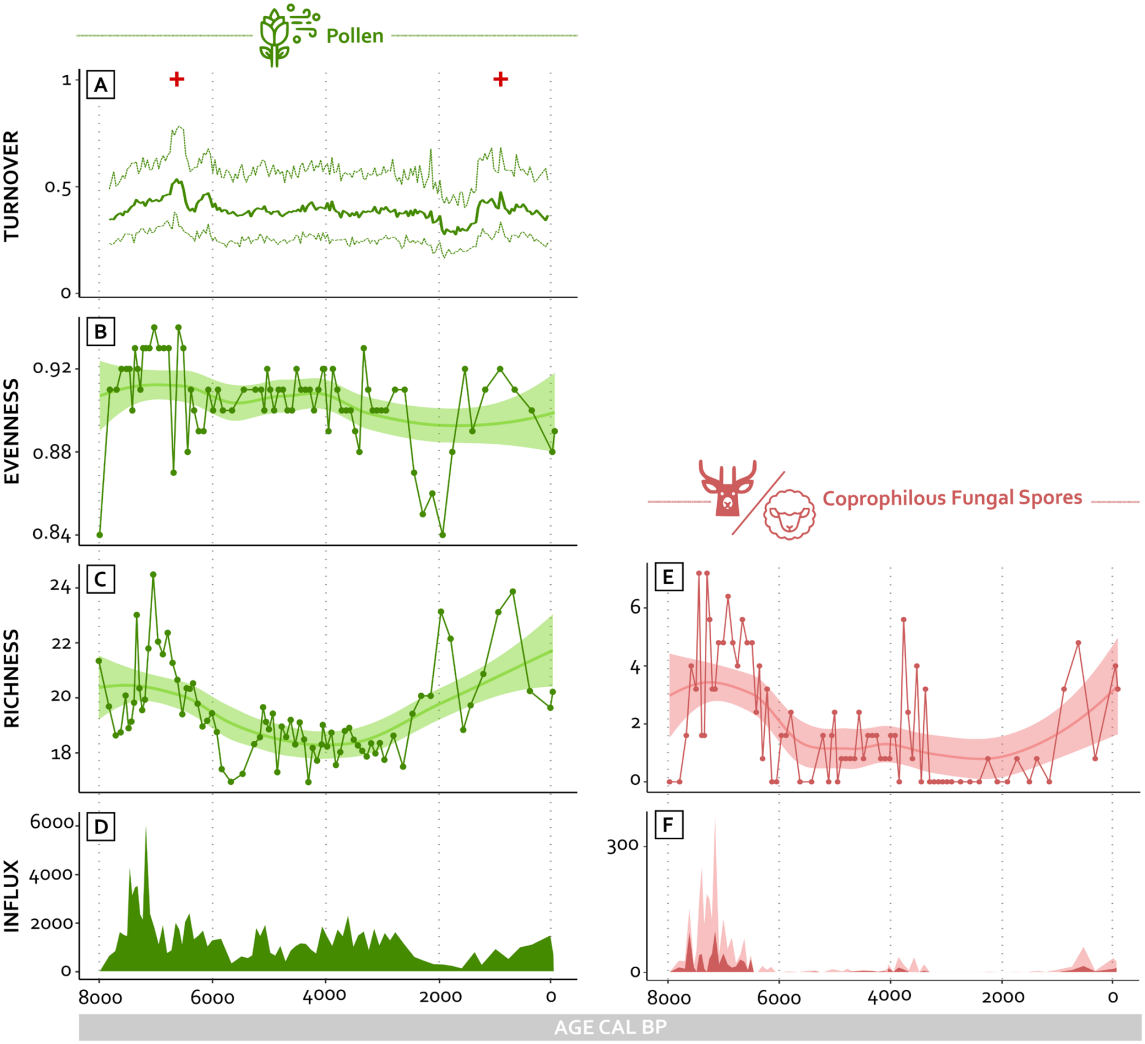
Pollen and coprophilous fungal spores diversity metrics: (A) Palynological turnover (rate of change has been computed based on an algorithm [R package R‐Ratepol] for estimating rates of change for palaeoecological time series); (B) Palynological evenness (probability of interspecific encounters [PIE]); (C) the palynological richness (PRI) represents the α‐diversity corresponding to the expected number of taxa found in samples of equal size as estimated by rarefaction analysis and has been statistically detrended to control for the influence of evenness, following the method proposed by Colombaroli and Tinner (2013); (D) Palynological influx (# cm^−2^ year); (E) Coprophilous fungal spores richness (the specnumber function from the R package “vegan” was used because rarefaction analysis was not applicable due to the low influx); and (F) Coprophilous fungal spores influx (# cm^−2^ year). The light red represents all the coprophilous spores used for the study and the dark red section represents spore species well documented in the scientific literature as reliable indicators of herbivores density or pastoral activities. Details of species and references are provided in Table S3. Icons are from the noun project (https://thenounproject.com/).

Palynological turnover exhibits two pronounced peaks that correspond to major ecological transitions in the sequence (Figure 4A). The first peak aligns with the transition between Periods 1 and 2, around 6500 years cal. BP, reflecting a shift from open, fire‐prone habitats to more closed and less flammable forests, including beech stands. The second peak occurs at the transition between Periods 3 and 4, around 1000 years cal. BP, and marks a return to open landscapes associated with garrigue, grasslands, and green oak coppices. Evenness remains consistently high across the sequence, with values above 0.80 (Figure 4B), indicating a stable distribution of pollen taxa and sustained ecological heterogeneity over millennia. Detrended palynological richness shows substantial variation through time (Figure 4C), and increases during phases dominated by open habitats and high‐herbivore activity (Figure 4E,F). Pollen influx also varies markedly, with elevated values in periods characterized by low canopy cover and productive grassland environments (Figure 4D). Coprophilous fungal spore richness fluctuates across the sequence (Figure 4E), and spore influx shows distinct peaks during intervals associated with open habitats (Figure 4F).

**FIGURE 5:**
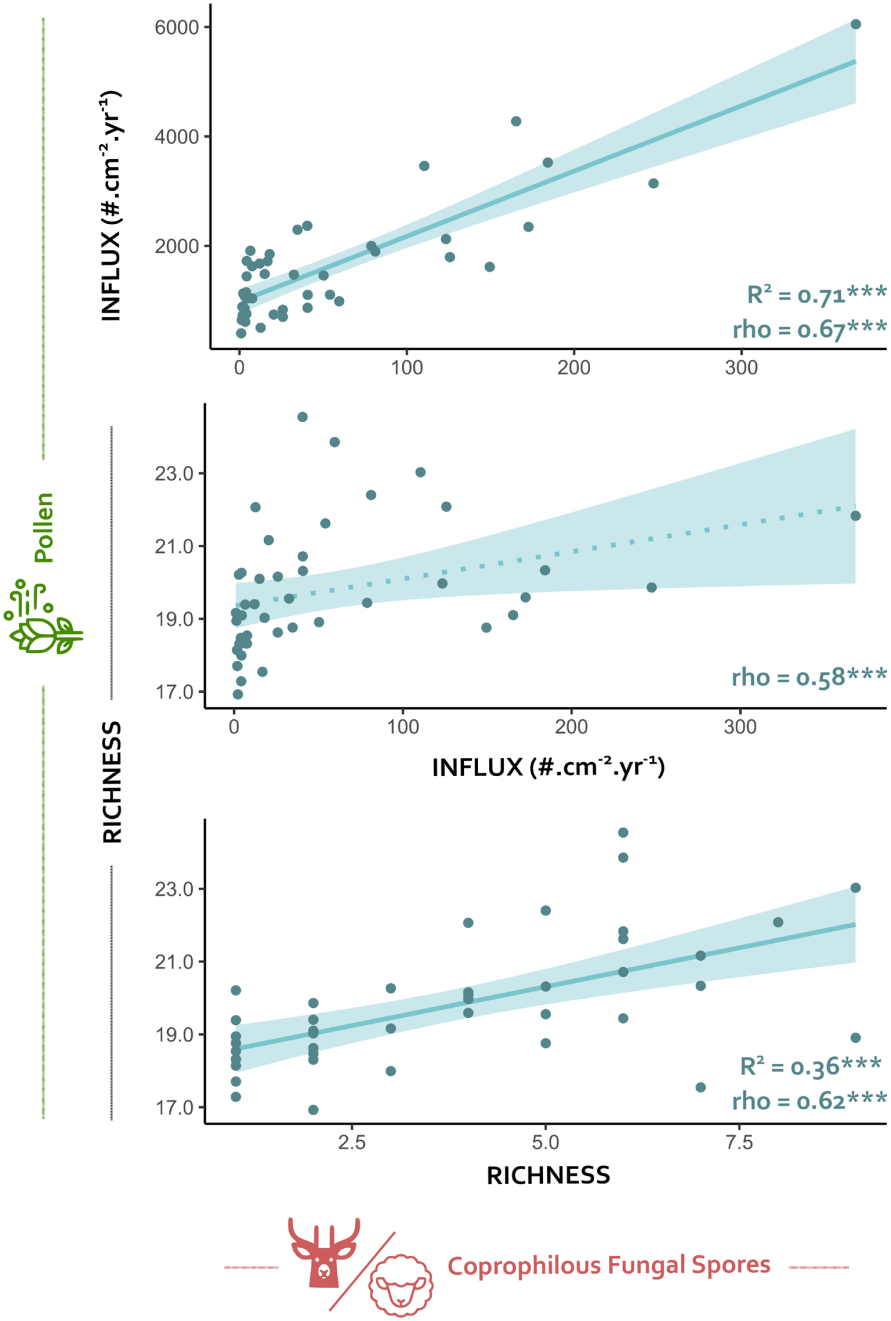
From top to bottom: palynological influx as a function of coprophilous fungal spores' influx. A spearman test highlighted significant positive correlation between these two variables (*p* value < 0.0001***, *r* = 0.67); palynological detrended richness as a function of coprophilous fungal spores' influx. A spearman test highlighted a significantly positive correlation between these two variables (*p* value < 0.0001***, rho = 0.58); and palynological detrended richness as a function of coprophilous fungal spores Richness. A spearman test highlighted a significantly positive correlation between these two variables (*p* value < 0.0001***, *r* = 0.62). A linear regression line was added to each panel for visualization purposes; in the first and third cases, the use of a linear model is justified by the normality of residuals.

Spearman's rank correlation tests reveal strong positive associations between pollen influx and coprophilous fungal spore influx (rho = 0.67, *p* < 0.0001), between palynological richness and spore influx (rho = 0.58, *p* < 0.0001), and between palynological richness and coprophilous fungal spore richness (rho = 0.62, *p* < 0.0001) (Figure 5). For the pollen–spore influx relation, the regression model yielded a high coefficient of determination (*R* ^2^ = 0.71, *p* < 0.0001), with residuals meeting normality assumptions. The relation between palynological richness and coprophilous fungal spore richness showed a moderate linear association (*R* ^2^ = 0.36, *p* < 0.0001), also supported by normally distributed residuals. In contrast, the regression between palynological richness and herbivore density through spore influx was not retained for interpretation due to non‐normality of residuals, and is shown for illustrative purposes only.

## 4 Discussion

### 4.1 Habitat Combustibility Dynamics in a Changing Environment

Open Mediterranean landscapes such as grasslands and garrigue are generally more combustible under typical summer‐dry conditions, whereas closed habitats like white oak coppices and beech forests tend to reduce fire propagation due to higher canopy cover, shaded microclimates, and greater fuel moisture, although under extreme drought or wind events, these forests may also reach high combustibility levels (e.g., Dimitrakopoulos and Papaioannou 2001; Fernandes et al. 2008; Ganteaume et al. 2011; Lestienne et al. 2023; Trabaud 1971). This structural contrast is well illustrated in the Crau Plain. These shifts reflect a complex interplay between climate variability, fire regimes, herbivore pressure, and human activities. Fire is recognized as a key ecological driver in Mediterranean systems (He et al. 2019), and its influence on the Crau Plain is evident in the higher combustibility of habitats observed before 6500 years cal. BP and after 1000 years cal. BP. During these periods marked by warmer and drier conditions (Colombaroli and Tinner 2013), fire‐adapted species thrived in open and dry environments (Roberts et al. 2019). Fire acts as a selective pressure favoring pyrophytic species but also plays a critical role in shaping plant communities composition by suppressing fire‐sensitive species and promoting open vegetation (Pausas and Keeley 2009). This process leads to a mosaic of habitats that supports high biodiversity, particularly in grassland and shrubland ecosystems (Roberts et al. 2019). Conversely, phases of reduced fire activity illustrated by lower combustibility from 6500 years cal. BP coincided with wetter and colder climatic conditions (Magny et al. 2007). These phases favored the expansion of closed‐canopy habitats such as white oak coppices and beech forests, which in turn suppressed fire spread (Morales‐Molino et al. 2021). This combustibility index is consistent with more recent approaches assessing vegetation combustibility or equivalent community‐level fire propensity metrics (e.g., Dimitrakopoulos and Papaioannou 2001; Ganteaume et al. 2011; Pellizzaro et al. 2007), which all confirm the marked contrasts in fuel behavior among Mediterranean vegetation types.

Human influence emerged as a key driver of habitat composition and fire regimes, acting either to suppress fires by reducing fuel through agriculture and mammal herbivory, or to promote them through deliberate burning for land clearance or pasture renewal (Costafreda‐Aumedes et al. 2018; Lestienne, Hély, et al. 2020). Before 6000 years cal. BP, we hypothesize that herbivory was primarily driven by wild herbivores, as no archaeological or palaeoecological evidence currently supports the presence of pastoral activity in the Crau Plain during this early period. The subsequent decline in wild herbivore densities (Figure 3) likely reflects early hunting pressure and land‐use changes (Etienne et al. 2013). Then, mammal herbivory by domestic livestock transformed the landscape by promoting open habitats such as grasslands and garrigue/heathland (Berger et al. 2019; Fyfe et al. 2015). This transformation increased the prevalence of highly combustible environments, particularly after the introduction of these pastoral systems (Archibald et al. 2013). In transitional periods, shifts in habitat composition highlight the interplay between natural and anthropogenic forces. For example, the gradual replacement of beech and white oak coppice by green oak coppice and pine forest reflects a combination of human‐driven deforestation, selective tree clearance, and climatic fluctuations (Roberts et al. 2019). These habitats are less fire‐resistant and mark the adaptive response of Mediterranean ecosystems to changing environmental pressures. The recent landscape dynamics, characterized by forest expansion following land abandonment (Berger et al. 2019), further demonstrate the evolving relationships between humans and the environment.

The recent increase in forest cover, dominated by combustible species such as green oak coppice and pine, appears to have increased fire risks by accumulating dense woody biomass that can fuel severe fires under dry conditions (Ertugrul 2019; Lestienne et al. 2022). This trend confirms the unique situation of the area as of today, which reveals the necessity for stakeholders to account for ecological drivers such as both herbivores and human land use to mitigate future fire hazards (Carrión et al. 2003; Henne et al. 2013). In this context, our results suggest that in addition to fire and land‐use changes, herbivory may have played a significant role in shaping habitat structure and diversity in the Crau Plain.

### 4.2 Herbivores Have Promoted High Plant Diversity

Although the respective impacts of fire, mammal herbivory, and land use are difficult to disentangle, our data indicate that herbivory functioned as a complementary force in sustaining vegetation openness and diversity within this specific Mediterranean lowland ecosystem. Contrary to the common perception that herbivores reduce diversity through over mammal herbivory or degradation, our data suggest that high‐herbivore presence, especially during open habitat phases, emerges as a key ecological driver of biodiversity, fostering both species richness (Figure 3) and heterogeneity (Figure 2) across Mediterranean landscapes (Cao et al. 2024; Filazzola et al. 2020). Mammal herbivory reduces woody plant dominance, preventing forest encroachment and enabling diverse plant communities to thrive (Bond and Keeley 2005; Papanikolaou et al. 2011). Despite significant changes in habitats and combustibility, community evenness remains relatively stable throughout millennia (Figure 3), indicating that landscape dynamics did not lead to homogenization. Palynological richness reaches its peaks in open, high‐herbivore‐density landscapes during the warm, dry climatic phase (Colombaroli and Tinner 2013) and periods of high combustibility (Figure 2), highlighting the role of herbivores in sustaining diverse grasslands and shrublands in the Crau Plain (Pausas and Verdú 2005). During these periods, palynological richness values were approximately 20% higher than during forested phases (Figure 3). In Mediterranean ecosystems, where a large part of plant diversity is associated with open and light‐demanding species, forest densification and canopy closure reduce light availability and limit the establishment of herbaceous taxa, resulting in a homogenization of plant communities (Pausas et al. 2004; Pausas and Keeley 2009). In the late Holocene, the gradual establishment of livestock mammal herbivory in the region contributed to reopening the landscape, increasing herbivore pressure and supporting a recovery of plant diversity. Mammal herbivory by domestic livestock plays a dual role: it maintains open habitats by limiting fuel accumulation, and contributes to creating a structurally heterogeneous landscape that can act as a barrier to fire propagation. While closed forests may provide other ecological benefits such as carbon storage and microclimate buffering (Birks and Tinner 2016; Bonan 2008; Shugart et al. 2010), our results highlight that, in Mediterranean lowland systems, maintaining a mosaic with open habitats is key to supporting biodiversity and reducing fire risk. In contrast, the recent decline in mammal herbivory intensity has promoted the accumulation of combustible biomass and facilitated woody species encroachment, thereby increasing the risk and continuity of severe fires (Bond and Keeley 2005). This highlights the complex interplay between herbivore density, vegetation structure, and fire regimes, where mammal herbivory historically contributed to maintaining open, fire‐resilient landscapes (Carmona et al. 2012; Papanikolaou et al. 2011).

Peaks in turnover during transitional phases suggest that vegetation responded rapidly to shifts in disturbance regimes, especially changes in herbivore pressure and habitat openness. These abrupt changes coincide with periods of restructuring in plant communities, highlighting the dynamic role of herbivory in shaping not only diversity levels but also the tempo and direction of ecological change. This goes beyond the maintenance of open landscapes and suggests that herbivores contributed to driving vegetation trajectories during key ecological transitions (Lestienne, Jouffroy‐Bapicot, et al. 2020; Sandom et al. 2014; Smit and Coetsee 2019). Our results show that herbivores have acted as long‐term regulators of biodiversity and vegetation structure, complementing the role of fire in sustaining open, diverse, and fire‐resilient ecosystems. By limiting the dominance of competitive species, they promote herbaceous communities that, while more flammable, tend to support less severe fires. This dual effect enhances species coexistence and contributes to a more resilient fire regime (Keeley 2009). These mechanisms are consistent with patterns observed across Mediterranean landscapes, notably in the Iberian Peninsula, where mammal herbivory has maintained species‐rich grasslands and prevented shrub encroachment (Blondel 2010; Pausas and Keeley 2009). Our findings extend these observations by highlighting that wild herbivores have contributed to biodiversity and habitat heterogeneity throughout the last millennia.

This pattern suggests that fire, together with herbivory, contributed to maintaining open and diverse landscapes through time. In this sense, fire can be considered a positive ecological force when it occurs within a mosaic of habitats and under the regulation of herbivore activity. However, this should not be interpreted as advocating uncontrolled, large‐scale wildfires, which have severe ecological and societal costs. Rather, it emphasizes the role of controlled or patchy fire regimes, often of low to moderate intensity, in sustaining biodiversity (e.g., Dupuy et al. 2020; Pausas and Keeley 2009).

### 4.3 Conservation Implications

Our findings highlight that herbivory has historically played a key role in maintaining open, diverse, and fire‐resilient habitats in Mediterranean ecosystems. However, the recent decline of traditional mammal herbivory practices, a widespread trend across southern Europe, has already led to the expansion of dense and homogeneous vegetation such as green oak coppice and garrigue (Otero et al. 2015). These habitats are less diverse, accumulate more combustible biomass and are more vulnerable to large, high‐intensity wildfires (Romero‐Díaz et al. 2017). This transition, already documented in several Mediterranean regions over the past century, is likely to accelerate under continued land abandonment and climate warming (Dupuy et al. 2020; Pausas and Keeley 2009).

If unaddressed, the loss of open habitats may lead to long‐term declines in biodiversity, reduced habitat heterogeneity and increasingly extreme fire regimes, with potentially irreversible consequences for some ecosystems (Papanikolaou et al. 2011; Romero‐Díaz et al. 2017). Yet this trajectory is not inevitable. Our results suggest that reintroducing or maintaining extensive herbivory could serve as an effective nature‐based solution to reduce fuel loads, promote landscape heterogeneity and preserve both biodiversity and fire resilience. Integrating these ecological insights into conservation and land management strategies is essential to adapt Mediterranean landscapes to ongoing environmental change.

## 5 Conclusion

This study underscores the intricate interplay between fires and herbivores in shaping Mediterranean landscapes and sustaining biodiversity. Fire has historically played a pivotal role in maintaining open habitats (Pausas 2004) and favoring fire‐adapted species (Pausas and Keeley 2009). Herbivores (both wild and human‐managed through pastoral practices) emerge as a complementary ecological force, limiting the encroachment of woody species, fostering ecological heterogeneity, and enhancing habitat resilience (Blondel 2010; Pausas and Verdú 2005). These actions limit fire intensity and propagation of the flame in the landscape. The trends observed during the late Holocene highlight the dual influence of natural processes and human activities, particularly mammal herbivory by both wild fauna and livestock, in preserving the mosaic of Mediterranean habitats (Berger et al. 2019; Roberts et al. 2019). However, the recent decline in mammal herbivory intensity and the associated expansion of closed habitats underscore the urgent need for proactive ecosystem management. Reintroducing sustainable mammal herbivory practices, coupled with strategies to mitigate woody vegetation encroachment, could play a crucial role in preserving the ecological balance, biodiversity, and diminishing severe fires of Mediterranean ecosystems (Carmona et al. 2012; Papanikolaou et al. 2011). These findings emphasize the importance of integrating fire and herbivore dynamics into holistic conservation and land‐use planning, particularly in the context of accelerating climate change and anthropogenic pressures. By embracing such integrated approaches, we can ensure the long‐term sustainability and resilience of these unique landscapes.

## Author Contributions

**Marion Lestienne:** conceptualization (equal), data curation (equal), formal analysis (lead), methodology (equal), validation (equal), visualization (lead), writing – original draft (lead), writing – review and editing (lead). **Pauline Saurat:** conceptualization (supporting), formal analysis (supporting), methodology (equal). **Christel Vidaller:** conceptualization (supporting), data curation (supporting), formal analysis (supporting), methodology (supporting), writing – review and editing (supporting). **Gwendal Mouden:** conceptualization (supporting), formal analysis (supporting), methodology (supporting). **Andy Hennebelle:** conceptualization (supporting), formal analysis (supporting), methodology (supporting), writing – original draft (supporting). **Lisa Bajolle:** data curation (equal), formal analysis (supporting), writing – original draft (supporting). **Bérangère Leys:** conceptualization (equal), data curation (equal), formal analysis (equal), funding acquisition (lead), investigation (equal), methodology (equal), project administration (equal), supervision (lead), validation (equal), visualization (supporting), writing – original draft (supporting), writing – review and editing (supporting).

## Conflicts of Interest

The authors declare no conflicts of interest.

## Supporting information


**Appendix S1:** ece372534‐sup‐0001‐AppendixS1.docx.

## Data Availability

The data supporting the results of this study are available in a Figshare repository : Lestienne, Marion (2025). Raw Data—Lestienne et al.—Ecology and Evolution. Figshare. Dataset. https://doi.org/10.6084/m9.figshare.29327189.v1.
