# Supplementary material for: Fire and Herbivory as Architects of Mediterranean Biodiversity
Source: Ecol Evol. 2025 Nov 20;15(11):e72534. doi: 10.1002/ece3.72534 (PMC12634060; doi:10.1002/ece3.72534)
Supplement: Supplementary file 1 — Appendix S1: ece372534‐sup‐0001‐AppendixS1.docx. [file ECE3-15-e72534-s001.docx]

- Supplementary materials -

Fire and Herbivory as Architects of Mediterranean Biodiversity

**
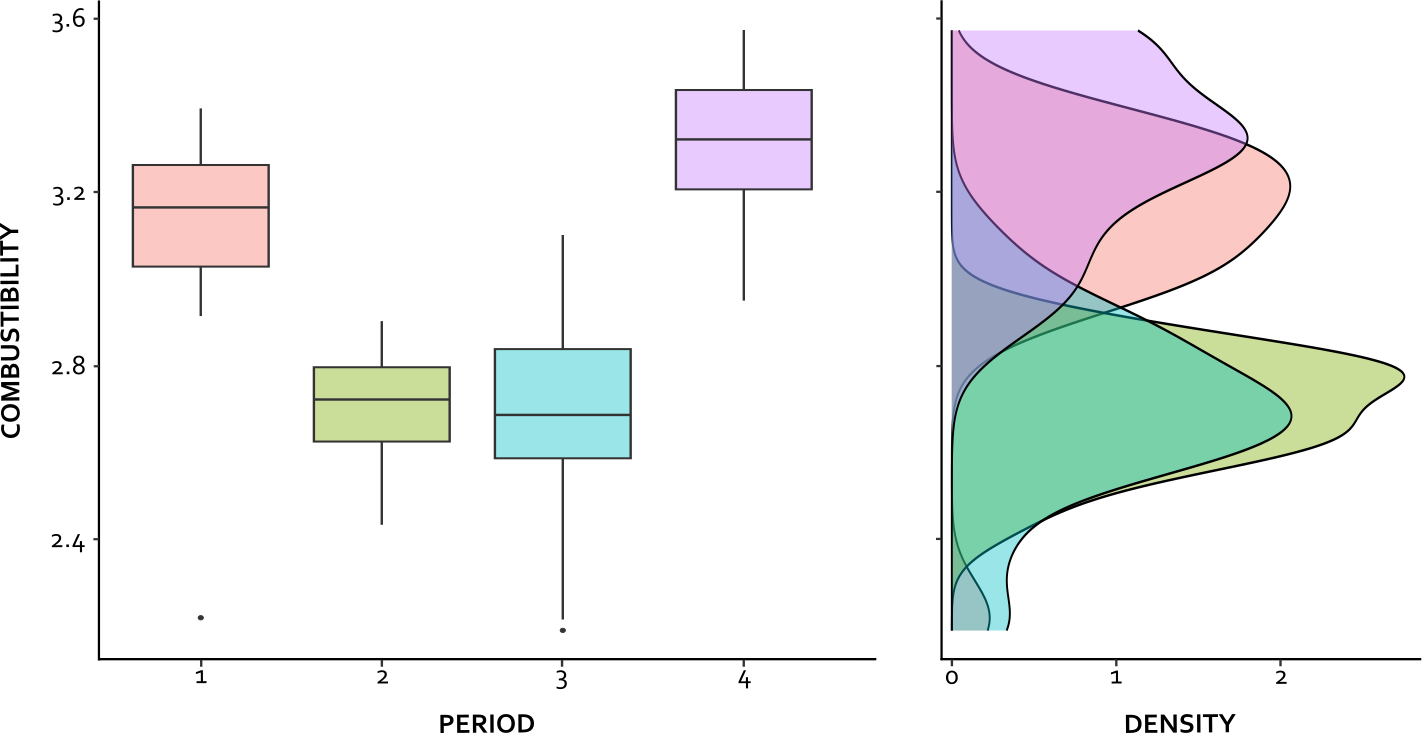
**

**Figure S1 -** Mean combustibility and distribution across periods. The mean combustibility per sample was calculated as a weighted average, using the formula:

Mean combustibility = $\frac{\boldsymbol{\sum(}\boldsymbol{influx}\left( \boldsymbol{i} \right)\boldsymbol{* combustibility}\left( \boldsymbol{i} \right)\boldsymbol{)}}{\boldsymbol{total influx}}$

where *i* corresponds to the habitat type. Significant differences between periods were determined using a non-parametric Mann-Whitney U test. Periods 1 and 2, 1 and 3, 2 and 4, 3 and 4 are significantly different (respectively: p-value = 1.754e^-08^ ; p-value = 1.004e^-08^; p-value = 1.281e^-06^; p-value = 1.677e^-06^). Periods 1 and 4, 2 and 3 are not significantly different (respectively: p-value = 0.1036; p-value = 0.9324).


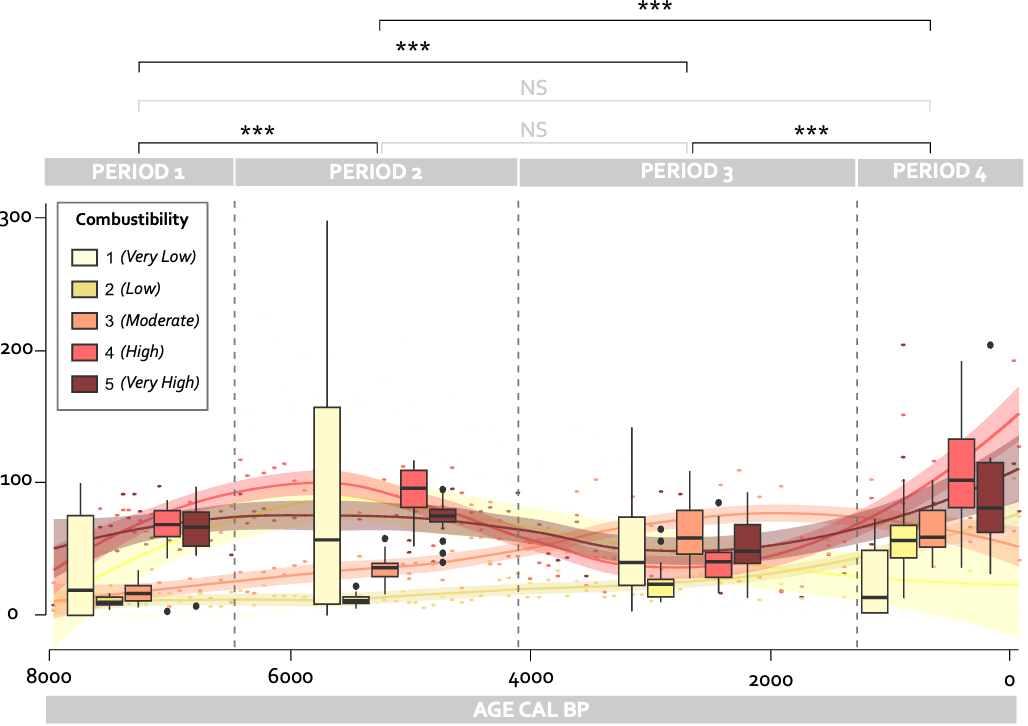


**Figure S2 -** Changes in habitat combustibility across the four significant different periods, as inferred from pollen data (#) and grouped according to Trabaud's (1971) habitat classification (*1*). Boxplots represent the distribution of combustibility classes (1: Very Low, 2: Low, 3: Moderate, 4: High, 5: Very High) for each period, with median values, interquartile ranges, and outliers shown. Colored ribbons highlight trends in combustibility over time, with darker shades corresponding to higher combustibility classes. Significant differences between periods were determined using a non-parametric Mann-Whitney U test. Periods 1 and 2, 1 and 3, 2 and 4, 3 and 4 are significantly different (respectively: p-value = 1.754e^-08^ ; p-value = 1.004e^-08^; p-value = 1.281e^-06^; p-value = 1.677e^-06^). Periods 1 and 4, 2 and 3 are not significantly different (respectively: p-value = 0.1036; p-value = 0.9324).

**Table S1** - Accelerator mass spectrometry radiocarbon dating of macroremains and of bulk sediments. The median depth and calibrated dates are used for the construction of the depth-age model, which extend from the first to the last sediment core sample analyzed from 0 to 3.1m of sediment). Three dates were not used to make the age model (in red) because they correspond to unreliable bulk sediment dates.

| Sample ID | Material | Lab. No. | Age | Methods | Age cal. BP. (2σ) | |
| --- | --- | --- | --- | --- | --- | --- |
| 2A-1B-101 | macroremains | Poz-164674 | 6020 ± 40 BP | AMS | | 5980-6060 |
| 2B-1B-35.5 | macroremains | Poz-165279 | 4475 ± 35 BP | AMS | | 4440-4510 |
| 2B-1B-50 | macroremains | Poz-165280 | 4550 ± 35 BP | AMS | | 4515-4585 |
| 2B-1B-63 | macroremains | Poz-164675 | 5230 ± 40 BP | AMS | | 5190-5270 |
| 2B-1B-95 | macroremains | Poz-164676 | 5600 ± 40 BP | AMS | | 5560-5640 |
| 2A-1A-20 | bulk | Poz-171849 | 1115 ± 30 BP | AMS | | 1080-1150 |
| 2A-1A-60 | bulk | Poz-171850 | 2645 ± 30 BP | AMS | | 2610-2680 |
| 2A-1A-100 | bulk | Poz-171851 | 4330 ± 35 BP | AMS | | 4295-4365 |
| 2A-1B-136 | bulk | Poz-171852 | 5545 ± 35 BP | AMS | | 5510-5580 |
| 2A-1B-176 | bulk | Poz-171854 | 5850 ± 40 BP | AMS | | 5810-5890 |
| 2B-1B-258 | bulk | Poz-171855 | 6590 ± 40 BP | AMS | | 6550-6630 |
| 2B-1B-288 | bulk | Poz-171856 | 6450 ± 40 BP | AMS | | 6410-6490 |

**Table S2 -** Combustibility classes, pollen percentages, pollen taxa and associated species for Mediterranean habitats, providing insights into vegetation dynamics and fire behavior. Pollen taxa contributing less than 1% of the total pollen record are written in grey. A total of 72.48% of the identified pollen has been attributed to a Trabaud habitat, classified by combustibility (1: Very Low to 5: Very High) based on vegetation flammability and fire spread potential. Total pollen percentages represent each habitat’s abundance over the entire sequence, while maximum percentages highlight peak dominance in specific levels. Key habitats include Beech Forest (e.g., *Fagus*), White Oak Coppice (e.g., *Quercus pubescens*), Green Oak Coppice (e.g., *Quercus* *ilex*), Pine Forest (e.g., *Pinus*), Garrigue/Heathland (e.g., *Thymus*, *Calluna*), and Grassland (Poaceae, Fabaceae). An "Other" category encompasses 27.52% of the pollen, representing diverse taxa not tied to a Trabaud habitat, including riparian or floodplain vegetation (e.g., *Alnus*, *Abies*, *Ulmus*), which fall outside the habitats defined by Trabaud (*1*).

| Habitat | Combustibility | % total | % max | Pollen taxa | Associated species |
| --- | --- | --- | --- | --- | --- |
| Beech forest | 1 | 2.48 | 8.92 | *Fagus* (FAGACEAE) | *Fagus sylvatica* |
| White oak coppice | 1 | 23.28 | 28.57 | *Acer* (SAPINDACEAE)  *Corylus* (BETULACEAE)  *Mercurialis*-type (EUPHORBIACEAE)  *Pistacia* (ANACARDIACEAE)  *Quercus* *pubescens*/*robur*-type (FAGACEAE) | *Acer monspessulanum*  *Corylus avellana*  *Mercurialis perennis*  *Pistacia* sp.  *Quercus pubescens* |
| Green oak coppice | 2 | 4.75 | 1.51 | CAPRIFOLIACEAE  *Erica*-type (ERICACEAE)  *Hedera* (ARALIACEAE)  *Lonicera*-type (CAPRIFIOLACEAE)  *Phillyrea* (OLEACEAE)  *Quercus* *cerris*-type (FAGACEAE)  *Quercus* *ilex*/*coccifera*-type (FAGACEAE)  ROSACEAE  *Stachys*-*type* (LAMIACEAE) | *Lonicera implexa*  *Erica sp., Arbutus unedo*  *Hedera helix*  *Lonicera implexa*  *Phillyrea angustifolia*  *Quercus sp.*  *Quercus ilex, Quercus coccifera*  *Rubus ulmifolius*  *Stachys officinalis* |
| Pine forest | 3 | 9.99 | 3.36 | *Pinus* (PINACEAE) | *Pinus halepensis* |
| Garrigue / Heathland | 4 | 15.99 | 23.38 | CUPRESSACEAE  CYPERACEAE  *Calluna*-type (ERICACEAE)  EUPHORBIACEAE  LAMIACEAE  LILIACEAE  *Olea* (OLEACEAE)  *Plantago* *coronopus*-type (PLANTAGINACEAE)  *Plantago* *tenuiflora*-type (PLANTAGINACEAE)  *Plantago*-type (PLANTAGINACEAE)  RANUNCULACEAE  *Thymus*-type (LAMIACEAE) | *Juniperus communis, Juniperus oxycedrus*  *Carex halleriana, Carex humilis*  *Calluna vulgaris*  *Euphorbia nicaeensis*  *Teucrium sp., Rosmarinus sp., Lavandula sp., Stachys sp.*  *Asparagus sp., Asphodelus sp., Aphyllanthes sp., Smilax sp.*  *Olea sp., Phyllyrea sp.*  *Plantago lanceolata*  *Plantago lanceolata*  *Plantago lanceolata*  *Ranunculus bulbosus*  *Thymus sp.* |
| Grasssland | 5 | 14.98 | 22.59 | APIACEAE  CONVOLVULACEA  *Cerealia*-type (POACEAE)  FABACEAE  *Medicago*-type (FABACEAE)  *Mentha*-type (LAMIACEAE)  *Ononis*-type (FABACEAE)  POACEAE  *Polygonum* (POLYGONACEAE)  *Sanguisorba* minor-type (ROSACEAE)  *Triticum*-type (POACEAE) | *Bupleurum sp., Eryngium campestre*  *Convolvulus cantabricus*  *Brachypodium sp., Bromus sp., Poa sp., Koeleria sp.*  *Anthyllis vulneraria, Lotus corniculatus, Trifolium stellatum*  *Medicago lupulina*  *Mentha sp.*  *Ononis sp.*  *Brachypodium sp., Bromus sp., Poa sp., Koeleria sp.*  *polygonum bistorta*  *Sanguisorba minor*  *Triticum sp.* |
| Other | / | 28.52 | 30.48 | *Abies* (PINACEAE)  *Alchemilla* (ROSACEAE)  *Alnus* (BETULACEAE)  *Ambrosia*/*Xanthium*-type (ASTERACEAE)  *Anthriscus*-type (APIACEAE)  *Artemisia* (ASTERACEAE)  *Aruncus* (ROSACEAE)  Asteroideae (ASTERACEAE)  BRASSICACEAE  *Betula* (BETULACEAE)  *Buxus* (BUXACEAE)  CHENOPODIACEAE  *Cannabis* (CANNABACEAE)  *Carpinus*/*Ostrya*-type (BETULACEA)  *Cedrus* (PINACEAE)  *Celtis* (CANNABACEAE)  *Centaurea* (ASTERACEAE)  *Cerastium*-type (CARYOPHYLLACEAE)  Cichorioidae (ASTERACEAE)  *Cirsium*-type (ASTERACEAE)  *Cistus* (CISTACEAE)  *Cotinus* (ANACARDIACEAE)  DIPSACACEAE  *Draba*-type (BRASSICACEAE)  *Ephedra* *fragilis*-type (EPHEDRACEAE)  *Fraxinus* (OLEACEAE)  GENTIACEAE  *Gagea*-type (LILIACEAE)  *Galium* (RUBIACEAE)  *Geranium* (GERANIACEAE)  *Helianthemum* (CISTACEAE)  *Herniari*, *Paronychia*, *Illecebrum*-type (CARYOPHYLLACEAE)  *Hottenia* (PRIMULACEAE)  *Hypericum* (HYPERICACEAE)  *Juglans* (JUGLANDACEAE)  *Legum*-type (ERICACEAE)  *Ligustrum* (OLEACEAE)  *Linium* (LINACEAE)  *Lythrym* (MYTHRACEAE)  MALVACEAE  *Myrica* (MYRICACEAE)  *Odontite* (OROBANCHACEAE)  *Ornithogalum*-type (LILIACEAE)  *Paronychia*-type (CARYOPHYLLACEAE)  *Picea* (PINACEAE)  *Pimpinella*-type (APIACEAE)  *Platanus* (PLATANACEAE)  *Polycnemum*-type (AMARANTHACEAE)  *Popolus* (SALICACEAE)  *Potentilla* (ROSACEAE)  *Reseda*-type (RESEDACEAE)  *Rhamnus* (RHAMNACEAE)  *Rhus* (ANACARDIACEAE)  *Ribes*-type (GROSSULACEAE)  *Rumex* (POLYGONACEAE)  SCROPHULARIACEAE  *Salix* (SALICACEAE)  *Sammel*-gp (APIACEAE)  *Scabiosa*-type (CAPRIFIOLACEAE)  *Silene*-type (CARYOPHYLLACEAE)  *Taxus* (TAXACEAE)  *Thalictrum* (RANUNCULACEAE)  *Tilia* (MALVACEAE)  *Trifolium*-type (FABACEAE)  URTICACEAE  *Ulmus* (ULMACEAE)  *Veronica* (PLANGINACEAE)  *Vitis* (VITACEAE) |  |

**Table S3 -** List of coprophilous fungal spore taxa used in the analysis. Taxa highlighted in gray are those not commonly referenced in the scientific literature as indicators of herbivory or pastoralism. In contrast, well-documented taxa, such as *Sporormiella-type*, *Podospora-type*, *Sordaria-type*, *Ascobolus-type*, and *Ascodesmis-type*, are recognized as reliable indicators of herbivore presence or grazing activity. These spores alone do not allow for a clear distinction between wild and domestic herbivores. Our interpretations are supported by a bibliographic review and comparisons with existing literature to contextualize the results (*2*).

| Taxa | Max count | Reference(s) |
| --- | --- | --- |
| *Sporormiella*-type | 20 | (*3*–*7*) |
| *Podospora*-type | 8 | (*6*, *8*–*11*) |
| *Sordaria*-type | 5 | (*6*, *8*–*10*, *12*, *13*) |
| *Ascobolus*-type | 3 | (*6*, *8*–*10*, *14*, *15*) |
| *Ascodesmis*-type | 3 | (*8*–*10*, *16*, *17*) |
| *Coniochaeta*-type | 14 | (*6*, *7*, *18*) |
| *Apiosordaria*/*Cercophora*-type | 5 | (*6*, *7*, *13*, *19*, *20*) |
| *Chaetomium*-type | 3 | (*6*, *7*, *19*) |
| *Arnium*-type | 2 | (*6*, *21*, *22*) |
| Xylariales-type | 3 | (*4*, *6*, *7*, *19*) |
| *Delitschia*-type | 2 | (*4*, *6*, *23*) |
| *Neurospora*/*Gelasinospora*-type | 2 | (*6*, *7*, *23*) |

# SI references

1. L. Trabaud, Influence du feu sur les propriétés chimiques des couches superficielles d’un sol de garrigue. *Revue d’écologie et de biologie du sol* **27**, 383–394 (1990).

2. C. M. Lee, B. van Geel, W. D. Gosling, On the Use of Spores of Coprophilous Fungi Preserved in Sediments to Indicate Past Herbivore Presence. *Quaternary* **5**, 30 (2022).

3. J. L. Gill, J. W. Williams, S. T. Jackson, K. B. Lininger, G. S. Robinson, Pleistocene megafaunal collapse, novel plant communities, and enhanced fire regimes in North America. *Science* **326**, 1100–1103 (2009).

4. M. Lestienne, I. Jouffroy-Bapicot, D. Leyssenne, P. Sabatier, M. Debret, P.-J. Albertini, D. Colombaroli, J. Didier, C. Hély, B. Vannière, Fires and human activities as key factors in the high diversity of Corsican vegetation. *The Holocene* **30**, 244–257 (2020).

5. I. Jouffroy-Bapicot, B. Vannière, V. Iglesias, M. Debret, J.-F. Delarras, 2000 Years of Grazing History and the Making of the Cretan Mountain Landscape, Greece. *PLOS ONE* **11**, e0156875 (2016).

6. A. Leal, X. Martínez-Blanco, Á. Beri, L. del Puerto, A combined catalog of non-pollen palynomorphs (NPPs) of fungal origin from soil and airborne samples of Uruguay. *Review of Palaeobotany and Palynology* **293**, 104488 (2021).

7. B. van Geel, V. Gelorini, A. Lyaruu, A. Aptroot, S. Rucina, R. Marchant, J. S. S. Damsté, D. Verschuren, Diversity and ecology of tropical African fungal spores from a 25,000-year palaeoenvironmental record in southeastern Kenya. *Review of Palaeobotany and Palynology* **164**, 174–190 (2011).

8. Y. Wei, G. Gao, D. Jie, E. N. van Asperen, L. Song, M. Meng, Z. Yang, N. Chen, J. Yu, Y. Li, Indication of coprophilous fungal spores for monitoring grazing intensity in the Horqin Sandy Land, Northern China. *Progress in Physical Geography: Earth and Environment*, 03091333241258892 (2024).

9. M. J. Richardson, Coprophilous fungi from Brazil. *Braz. arch. biol. technol.* **44**, 283–289 (2001).

10. A. Bell, *Dung Fungi: An Illustrated Guide to Coprophilous Fungi in New Zealand* (Victoria University Press, 1983).

11. B. Dietre, É. Gauthier, F. Gillet, Modern pollen rain and fungal spore assemblages from pasture woodlands around Lake Saint-Point (France). *Review of Palaeobotany and Palynology* **186**, 69–89 (2012).

12. H. Wei, R. Duan, Q. Xu, S. Yang, Q. Fan, G. Hou, Y. Du, Z. Qin, J. Gao, Fungal spore indicators of vegetation and highland pastoralism in modern topsoil and dung, eastern Tibetan Plateau. *Catena* **202**, 105231 (2021).

13. B. van Geel, A paleoecological study of holocene peat bog sections : based on the analysis of pollen, spores and macro- and microscopic remains of fungi, algae, cormophytes and animals /. *Review of Palaeobotany and Palynology* **25**, 1–120 (1978).

14. A. D. Parker, Associations between coprophilous ascomycetes and fecal substrates in Illinois. *Mycologia* **71**, 1206–1214 (1979).

15. A. A. Miyunga, “Morphological and molecular characterization of ascobolus and pilobolus fungi in wild herbivore dung in Nairobi National Park,” thesis, Egerton University (2015).

16. R. Ghosh, D. K. Paruya, K. Acharya, N. Ghorai, S. Bera, How reliable are non-pollen palynomorphs in tracing vegetation changes and grazing activities? Study from the Darjeeling Himalaya, India. *Palaeogeography, Palaeoclimatology, Palaeoecology* **475**, 23–40 (2017).

17. A. Pandey, S. Tripathi, S. K. Basumatary, “Non-Pollen Palynomorphs from the Late-Holocene Sediments of Majuli Island, Assam (Indo-Burma Region): Implications to Palaeoenvironmental Studies” in *Climate Change and Environmental Impacts: Past, Present and Future Perspective* (Springer, 2023), pp. 63–81.

18. A. G. Perrotti, E. Van Asperen, Dung fungi as a proxy for megaherbivores: opportunities and limitations for archaeological applications. *Vegetation History and Archaeobotany* **28**, 93–104 (2019).

19. C. Cugny, F. Mazier, D. Galop, Modern and fossil non-pollen palynomorphs from the Basque mountains (western Pyrenees, France): the use of coprophilous fungi to reconstruct pastoral activity. *Veget Hist Archaeobot* **19**, 391–408 (2010).

20. B. van Geel, J. Buurman, O. Brinkkemper, J. Schelvis, A. Aptroot, G. van Reenen, T. Hakbijl, Environmental reconstruction of a Roman Period settlement site in Uitgeest (The Netherlands), with special reference to coprophilous fungi. *Journal of Archaeological Science* **30**, 873–883 (2003).

21. A. G. Baker, S. A. Bhagwat, K. J. Willis, Do dung fungal spores make a good proxy for past distribution of large herbivores? *Quaternary Science Reviews* **62**, 21–31 (2013).

22. H. A. Raja, C. A. Shearer, Arnium gigantosporum, a new ascomycete species from fresh water in Florida. *Fungal Diversity* **22**, 219–225 (2006).

23. Md. F. Quamar, N. Stivrins, Modern pollen and non-pollen palynomorphs along an altitudinal transect in Jammu and Kashmir (Western Himalaya), India. *Palynology* **45**, 669–684 (2021).
